# Dynamic Compression of Chondrocyte-Agarose Constructs Reveals New Candidate Mechanosensitive Genes

**DOI:** 10.1371/journal.pone.0036964

**Published:** 2012-05-17

**Authors:** Carole Bougault, Elisabeth Aubert-Foucher, Anne Paumier, Emeline Perrier-Groult, Ludovic Huot, David Hot, Martine Duterque-Coquillaud, Frédéric Mallein-Gerin

**Affiliations:** 1 Université Lyon 1, Univ Lyon, CNRS, FRE 3310 – Dysfonctionnement de l'Homéostasie Tissulaire et Ingénierie Thérapeutique, IBCP, Lyon, France; 2 Transcriptomics and Applied Genomics, Institut Pasteur de Lille – Center for Infection and Immunity of Lille, U1019, UMR 8204, Lille, France; 3 CNRS UMR 8161, Institut de Biologie de Lille, Université de Lille Nord de France, Institut Pasteur de Lille/IFR142, Lille, France; University of Western Ontario, Canada

## Abstract

Articular cartilage is physiologically exposed to repeated loads. The mechanical properties of cartilage are due to its extracellular matrix, and homeostasis is maintained by the sole cell type found in cartilage, the chondrocyte. Although mechanical forces clearly control the functions of articular chondrocytes, the biochemical pathways that mediate cellular responses to mechanical stress have not been fully characterised. The aim of our study was to examine early molecular events triggered by dynamic compression in chondrocytes. We used an experimental system consisting of primary mouse chondrocytes embedded within an agarose hydrogel; embedded cells were pre-cultured for one week and subjected to short-term compression experiments. Using Western blots, we demonstrated that chondrocytes maintain a differentiated phenotype in this model system and reproduce typical chondrocyte-cartilage matrix interactions. We investigated the impact of dynamic compression on the phosphorylation state of signalling molecules and genome-wide gene expression. After 15 min of dynamic compression, we observed transient activation of ERK1/2 and p38 (members of the mitogen-activated protein kinase (MAPK) pathways) and Smad2/3 (members of the canonical transforming growth factor (TGF)-β pathways). A microarray analysis performed on chondrocytes compressed for 30 min revealed that only 20 transcripts were modulated more than 2-fold. A less conservative list of 325 modulated genes included genes related to the MAPK and TGF-β pathways and/or known to be mechanosensitive in other biological contexts. Of these candidate mechanosensitive genes, 85% were down-regulated. Down-regulation may therefore represent a general control mechanism for a rapid response to dynamic compression. Furthermore, modulation of transcripts corresponding to different aspects of cellular physiology was observed, such as non-coding RNAs or primary cilium. This study provides new insight into how chondrocytes respond to mechanical forces.

## Introduction

Articular cartilage is a highly specialised connective tissue in joints. Its main function is to provide a smooth, lubricated surface for articulation and to take up and distribute high loads. Its remarkable dimensional stability and mechanical properties are due to the composition of its extracellular matrix. The load-bearing function is based on the high osmotic pressure created by negatively charged glycosaminoglycans, which are predominantly aggrecan molecules. In addition, the fibrillar collagen network, mainly composed of type II collagen, provides the tissue with its tensile resistance. As the only cell type in articular cartilage, chondrocytes are entirely responsible for maintaining the metabolic balance of matrix proteins. Accordingly, it has been shown that mechanical forces affect chondrocyte metabolic activity (for a review, see [1]). More precisely, *ex vivo* and *in vitro* models of chondrocyte mechanobiology have generally shown that static compression inhibits the expression of cartilage matrix proteins whereas dynamic compression regimens enhance them [2]–[7].

In this context, mechanotransduction is the molecular process by which cells convert mechanical force into biochemical signalling. Little is currently known regarding the sequence of biochemical events that are involved in mechanotransduction and that eventually result in the modulation of the chondrocyte phenotype. It is therefore necessary to assess the signalling and regulatory pathways activated during mechanical signal transduction in chondrocytes. In this study, we employed microarray analysis to investigate the overall changes in chondrocyte gene expression in response to dynamic compression. We used a cell model system consisting of isolated mouse chondrocytes embedded within an agarose hydrogel. We have previously used these constructs to develop experimental procedures to analyse the effects of compression at the mRNA level (using reverse transcription-polymerase chain reaction experiments) and to determine the phosphorylation state of signalling molecules (using Western blotting) [8], [9]. Here, our study was designed to identify candidate genes involved in the early response of chondrocytes to compression.

**Figure 1:**
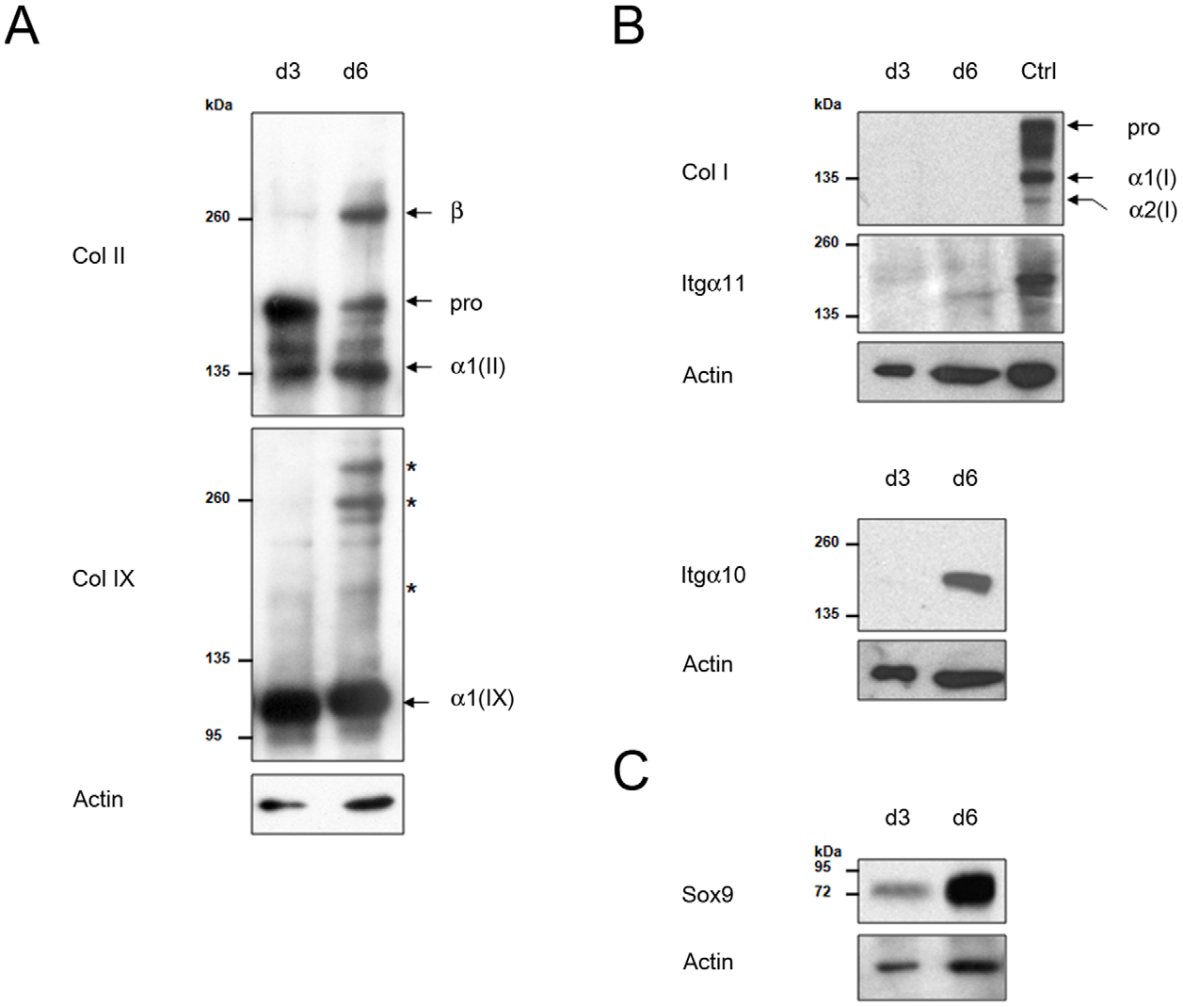
Chondrocytes embedded in agarose gel maintain a well-differentiated phenotype. Expression of extracellular matrix proteins, integrins and the Sox9 transcription factor were analysed on Western blots of chondrocytes cultured in 3D for 3 days (d3) or 6 days (d6). The presence or absence patterns of proteins at day 6 are representative of 3 independent experiments. A: Type II (Col II) and type IX (Col IX) collagens accumulate and become cross-linked by day 6. Procollagen II forms (pro) and mature collagen II chains [α1(II)] are present. Beta (β) indicates cross-linked α1(II) dimers. Collagen IX chains [α1(IX)] are present. Asterisks (*) indicate cross-linked collagens. B: Type I collagen (Col I) and α11 integrin (Itgα11) are not or only faintly detected, whereas the chondrocyte-specific α10 integrin (Itgα10) is present at day 6. Passaged chondrocytes cultured in monolayer were used as positive controls (Ctrl) for Col I and α11 integrin immunorevelations. Procollagen I (pro) and mature collagen I chains α1(I) and α2(I) are indicated. C: Sox9 chondrogenic transcription factor increases with the duration of culture.

Taken together, the results presented here indicate that the mitogen-activated protein kinase (MAPK) and the transforming growth factor (TGF)- β pathways are involved in the early response of chondrocytes to dynamic compression. The microarray analysis revealed that only 20 transcripts were modulated more than 2-fold. At a fold modulation threshold of 1.4, an extended list of candidate genes included 325 candidate mechanosensitive genes, of which 85% were down-regulated. This global down-regulation may indicate a general control mechanism for a rapid response to dynamic compression. Many of the observed modulated genes are known to be mechanosensitive in other biological contexts. In addition, modulation of genes or transcripts involved in various aspects of cellular physiology was observed. Our integrated analysis provides new molecular insight into how chondrocytes respond to mechanical forces.

**Figure 2:**
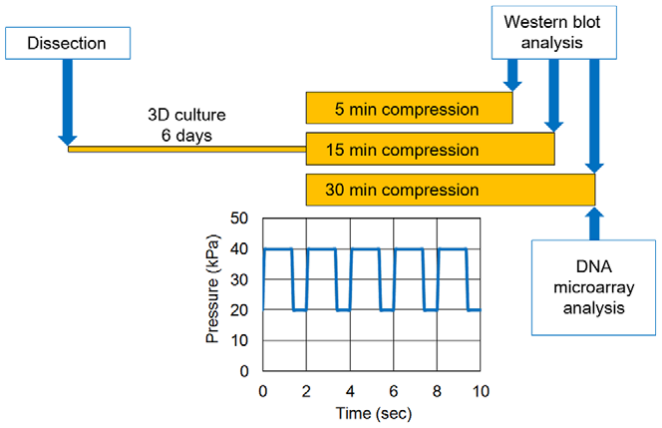
Experimental design and dynamic compression profile. Chondrocytes cultured in agarose for 6 days underwent dynamic compression using the FX-4000C Flexercell Compression Plus System (Flexcell International). Chondrocyte-agarose constructs underwent cyclical compression ranging from 20 kPa to 40 kPa at a frequency of 0.5 Hz for 5, 15 or 30 min. Signalling proteins were analysed by Western blot at each of the three time points. DNA microarray analysis was performed to compare 30 min-compression constructs to uncompressed constructs.

## Results

### Maintenance of the chondrocyte phenotype and cartilage-characteristic matrix deposition in an agarose hydrogel

To investigate the early effects of dynamic compression on gene expression of fully differentiated chondrocytes, we used a previously described cell model system [8], [9]. Briefly, mouse chondrocytes were embedded in agarose just after their isolation from cartilage and these chondrocyte-agarose constructs were cultured for 6 days to allow extracellular matrix deposition. Under these conditions, chondrocytes are viable and their proliferation was confirmed by an increase in DNA content (about 1.5 fold, data not shown). Furthermore, they maintain their round morphology and type II collagen and aggrecan accumulate at the cell periphery [8], [9]. Western blotting was used to obtain more detailed information on the matrix proteins and integrin receptors present in the chondrocyte-agarose constructs just before application of dynamic compression (Figure 1).

**Figure 3:**
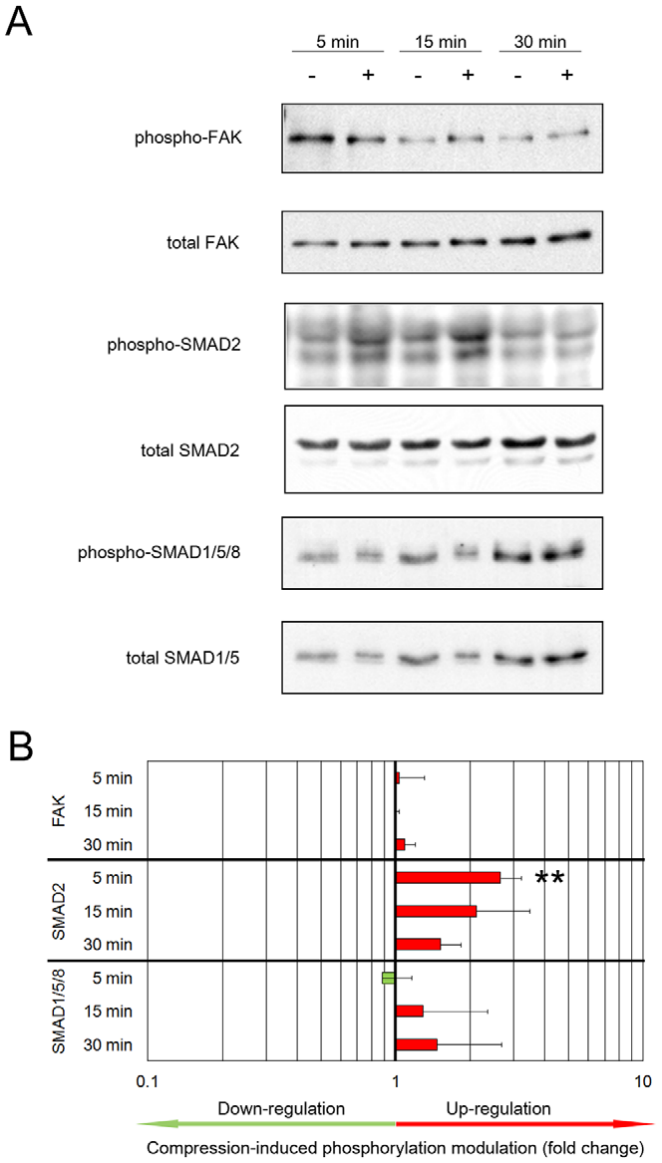
Smad2, but not Smad1/5/8 or FAK, is activated by compression in chondrocyte-agarose constructs. Chondrocytes cultured in agarose for 6 days underwent dynamic compression (+) or were not compressed (−) for the indicated times and the phosphorylation levels of FAK, Smad2 and Smad1/5/8 were analysed on Western blots. (A) Representative blots. (B) For FAK phosphorylation, densitometric analysis was performed on three (5 and 15 min) or two (30 min) independent experiments. For SMAD phosphorylation, densitometric analysis was performed on four (5 and 15 min) or three (30 min) independent experiments. For each protein, the ratio of the phospho-protein to the total protein was calculated and the value obtained for mechanically-induced phosphorylation was normalised to uncompressed controls. Bars represent the compression-induced phosphorylation modulation (mean +/− SD), with up-regulation in red and down-regulation in green (** p<0.01).

As expected, after 3 days of culture, chondrocytes synthesised type II collagen, but mainly in the procollagen form (Figure 1 Panel A). Fibrillar collagens such as type II collagen are synthesised as precursor forms that must be cleaved to produce the mature triple helical collagens capable of packing into fibrils (for a review, see [10]). After 6 days of culture, mature-form type II collagen was the predominant form and showed interchain covalent cross-links (Figure 1 Panel A). All the enzymes necessary for the post-translational maturation of collagen were therefore active in the 3D scaffolds. In addition, we investigated type IX collagen, which is a minor non-fibrillar collagen present in hyaline cartilage. Western blot analysis confirmed the presence of covalent cross-links between collagen molecules in the chondrocyte-agarose constructs after 6 days of culture (Figure 1 Panel A). To demonstrate the absence of critical proteins that could cause the chondrocytes to transduce mechanical signals in a non-characteristic way, we looked for type I collagen, the classical marker of fibroblasts and dedifferentiated chondrocytes. No type I collagen was detected in Western blots on the chondrocyte-agarose constructs, but it was detected in the positive controls, i.e. extracts of mouse chondrocytes cultured in monolayer (Figure 1 Panel B). Therefore, before the compression experiments, chondrocytes synthesise mature and cross-linked extracellular matrix components that are part of the typical collagen network in cartilage.

**Figure 4:**
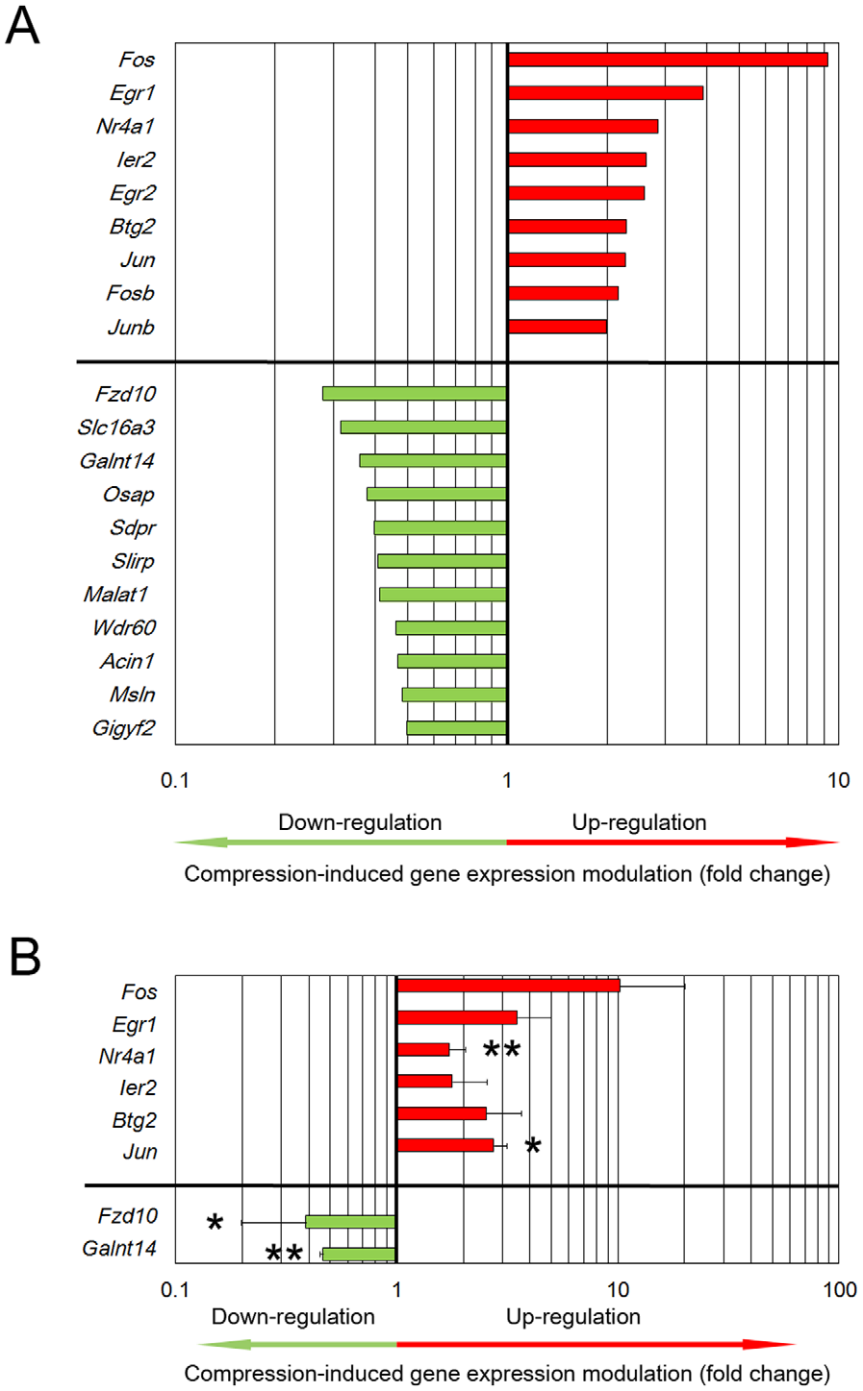
Identification of major candidate mechanosensitive genes. Gene expression levels of compressed samples were compared to uncompressed controls. (A) DNA microarray analysis was performed on four independent pairs of uncompressed/compressed experiments. Expression level differences were sorted to identify highly responsive genes (fold change >2), resulting in a list of 20 transcripts. Bars represent the fold change in gene expression upon compression, i.e. up-regulation (red) or down-regulation (green) (p<0.01). Exact modulation factors and associated p-values are detailed in Table 1. (B) Real-time PCR analysis on three independent experiments confirmed DNA microarray results for eight selected genes. Bars represent the compression-induced gene expression modulation (mean +/− SD), either up-regulation (red) or down-regulation (green) (* p<0.05, ** p<0.01).

Integrin transmembrane receptors connect the extracellular matrix to the intracellular cytoskeletal network and are expected to play an important role in cellular responses to mechanical forces. The main collagen-binding integrin on chondrocytes in cartilage is α10β1 integrin, whereas α11β1 integrin is more characteristic of mesenchymal tissues. Thus, α10 and α11 are good markers for evaluating the status of the chondrocyte phenotype [11]. Integrins, probably along with other surface proteins, were removed from the cell surface after enzymatic isolation of chondrocytes from cartilage (Figure 1 Panel B). α10 was re-expressed at the end of the culture period in agarose, whereas α11 could only be faintly detected. We also monitored another differentiation marker: Sox9, a transcription factor required for cartilage formation (Figure 1 Panel C). In mouse chondrocytes, high levels of Sox9 protein correlate with type II collagen synthesis and a well-differentiated phenotype, whereas dedifferentiated as well as hypertrophic chondrocytes lack Sox9 [12]. Thus, after a 6 day culture period, robust Sox9 expression together with α10 integrin expression further confirmed that chondrocytes were highly differentiated.

**Table 1:** Results from DNA microarray analysis: gene expression levels in compressed samples were compared to uncompressed control samples (fold change >2 and p-value >0.01).

RNA ID	PROTEIN ID	GENE NAME	FOLD CHANGE	ADJUSTED P-VALUE
		**UP-REGULATED GENES**		
NM_010234	Q6PCX9	Proto-oncogene protein c-fos;Fos	9.27	2E-04
NM_007913	Q9WVQ1	Early growth response protein 1;Egr1	3.90	1E-04
NM_010444	Q9DBG7	Nuclear receptor subfamily 4 group A member 1;Nr4a1	2.84	4E-04
NM_010499	P17950	Immediate early response gene 2 protein;Ier2	2.63	2E-05
NM_010118	Q9JLB2	Early growth response protein 2;Egr2	2.60	6E-03
NM_007570	Q04211	Protein BTG2;Btg2	2.28	5E-04
NM_010591	Q6SJQ0	Transcription factor AP-1;Jun	2.27	1E-02
NM_008036	P46935	Protein fosB;Fosb	2.16	4E-03
NM_008416	Q61136	Transcription factor jun-B;Junb	2.00	8E-04
		**DOWN-REGULATED GENES**		
NM_175284	Q149J3	Frizzled homolog 10;Fzd10	0.28	7E-07
NM_030696	Q8BL66	Monocarboxylate transporter 4;Slc16a3	0.31	2E-05
NM_027864	Q61468	Polypeptide N-acetylgalactosaminyltransferase 14;Galnt14	0.36	3E-05
NM_026358	Q8VI64	Ovary-specific acidic protein;Osap	0.38	8E-06
NM_138741	Q9D994	Serum deprivation-response protein;Sdpr	0.40	2E-03
AJ293625	Q9D8T7*	SRA stem-loop-interacting RNA-binding protein (mitochondrial);Slirp	0.41	4E-06
AK020134		Metastasis associated lung adenocarcinoma transcript 1 (non-coding RNA);Malat1	0.41	1E-03
AK032986	Q8BQ86	WD repeat-containing protein 60;Wdr60	0.46	7E-03
NM_023190	Q11011	Apoptotic chromatin condensation inducer in the nucleus;Acin1	0.47	3E-04
NM_018857	Q70KY4	Mesothelin, cleaved form;Msln	0.48	7E-05
NM_146112	Q6Y7W8	PERQ amino acid-rich with GYF domain-containing protein 2;Gigyf2	0.50	3E-03

In conclusion, the chondrocytes in our agarose model system were well-differentiated and did synthesise mature, cross-linked extracellular matrix components as well as integrins, before we applied dynamic compression.

**Figure 5:**
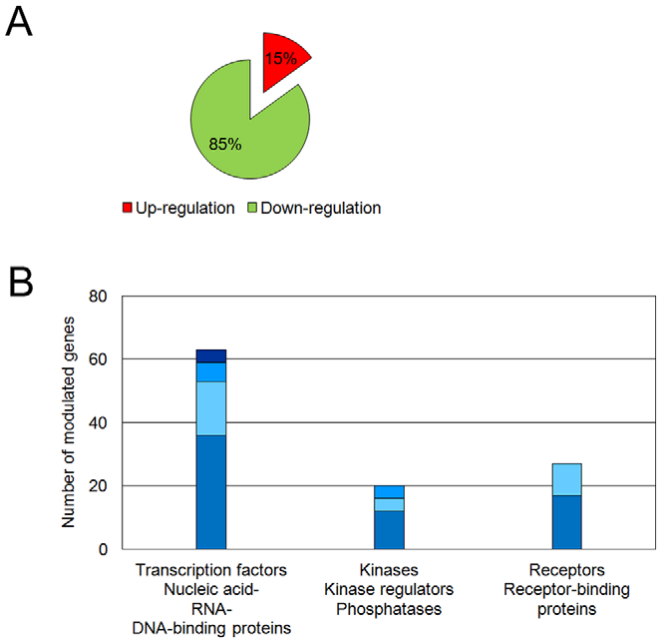
Analysis of the candidate mechanosensitive gene list. Gene expression levels of compressed samples were compared to uncompressed controls. DNA microarray analysis was performed on four independent pairs of compressed/uncompressed experiments. A list of 325 candidate genes was obtained by selecting transcripts with a fold change greater than 1.4 (p<0.01). (A) Distribution of up- and down-regulated transcripts. (B) Functional annotation highlighting genes involved in gene expression regulation and in signal transduction. Protein classes associated with modulated genes were pooled into three main groups: transcription regulation, phosphorylation cascade and receptor activity and the number of genes belonging to each protein class is shown. Within each group, protein classes are listed from most represented to least represented.

### Detection of MAPK pathway and Smad2 activation due to dynamic compression

The mitogen-activated protein kinase (MAPK) pathways involving ERK1/2 and p38 are implicated in chondrocyte mechanotransduction [13]–[15]. We therefore investigated MAPK activation in the chondrocyte-agarose constructs to select the appropriate duration of dynamic compression for the characterisation of mechanotransduction events. Chondrocyte-agarose constructs underwent dynamic compression for 5, 15 or 30 min (Figure 2) and Western blots were used to examine phosphorylation levels of ERK1/2 and p38. ERK1/2 phosphorylation was observed primarily after 15 min of compression (average 2-fold increase when compared to uncompressed samples) but high variability in the phosphorylation rates impaired the statistical significance of the results (Data S1). Following 5 min of compression, p38 phosphorylation stimulation was low but highly reproducible (p<0.05). After 15 min, this activation seemed stronger (3-fold induction), but again, the response was highly variable (Data S1).

**Table 2:** Primers used for real-time PCR analysis (reference gene: *Rpl13a*).

GENE NAME	PRIMER SEQUENCE	
*Rpl13a*	S	atccctccaccctatgacaa
	AS	gccccaggtaagcaaactt
*Fos*	S	gggacagcctttcctactacc
	AS	gatctgcgcaaaagtcctgt
*Egr1*	S	ccctatgagcacctgaccac
	AS	tcgtttggctgggataactc
*Nr4a1*	S	ctgtccgctctggtcctc
	AS	aatgcgattctgcagctctt
*Ier2*	S	ttgaatctcagggtcgaactc
	AS	ggtagtgaaacggccttgaa
*Btg2*	S	gcgagcagagactcaaggtt
	AS	ccagtggtgtttgtaatgatcg
*Jun*	S	agggacccatggaagttttt
	AS	tttttctaggagttgtcagattcaaa
*Fzd10*	S	tgctgcctgtgcataaactt
	AS	cccccaggaaagctctttag
*Galnt14*	S	tactatgcagctcggccttt
	AS	caggttcagcctgttctcaa

Focal adhesion kinase (FAK) is a non-receptor tyrosine kinase whose phosphorylation is generally detected within minutes after application of mechanical strain in a variety of cell types, including chondrocytes [16]. However, in our chondrocyte-agarose model system, we found no compression-induced increase in FAK phosphorylation (Figure 3).

Since TGF-β pathways activation was once reported as part of the cartilage response to mechanical strain [17], we analysed Smad phosphorylation with or without compression of the chondrocytes in agarose. Mechanical stimulation promoted Smad2 phosphorylation, mainly after 5 and 15 min of dynamic compression, whereas there were no differences in the phosphorylation levels of Smad1/5/8 between compressed and uncompressed samples (Figure 3). Results from four independent experiments revealed the relatively high intensity (2.5-fold induction) and the great reproducibility (p<0.01) of this early event of Smad2 activation (Figure 3).

Finally, the time-dependent activation of the MAPK and canonical TGF-β/Smad pathways demonstrated that the compression regimen we applied to our chondrocyte-agarose model system was sufficient to trigger a cellular response at the molecular level. These pathways, independently or in synergy, may induce changes in the expression of genes that are important in the early responses of chondrocytes to mechanical signals.

### Confirmation of the mechanosensitive character of members of the AP-1 transcription factor family and *Egr1*

Next, we undertook a microarray analysis to detect gene expression modulation in response to 30 min of dynamic compression. Observed gene expression regulation was thus putatively downstream the activation of the MAPK and TGF-β/Smad pathways observed after only 15 min of compression. An extensive microarray analysis was performed on four independent pairs of compressed and uncompressed experiments. We focused on highly responsive genes: a 2-fold change threshold for up- and down-regulation was applied (p<0.01). Of the 20 transcripts with a difference of 2-fold or more, 9 transcripts corresponded to up-regulated genes and 11 to down-regulated genes (Table 1 and Figure 4). Interestingly, 8 of the up-regulated genes were known transcription factors. The most responsive gene was *Fos*, with a compression-induced over-expression of about 9-fold (p<0.001) and *Egr1* showed a compression-induced up-regulation of about 4-fold (p<0.001). Furthermore, the concomitant stimulation of *Jun, Junb* and *Fosb*, which are all genes coding for members of the AP-1 transcription factor family, and *Egr1* has also been reported to occur as an early event in diverse models of compression for skeletal cells [18]–[22].

### Identification of new candidate mechanosensitive genes

In addition to those mentioned above, genes showing a difference of 2-fold or more with a p-value of less than 0.01 are listed in Table 1 (Figure 4, Panel A). Regarding the up-regulated transcription factor-encoding genes, *Egr2* and *Btg2* are members of the early growth response gene family and *Nr4a1* encodes a nuclear receptor. The last up-regulated gene in the list was *Ier2*, another early gene inducible by growth factors. Therefore, all up-regulated genes in this list are already known as “immediate early genes”.

The list of the down-regulated genes appeared more diversified. *Slc16a3*, *Galnt14*, *Osap* and *Slirp* code for proteins involved in cell metabolism, *Acin1* and *Msln* are genes related to cell death, *Sdpr/Cavin-2* encodes a caveolar protein, *Malat1* corresponds to a non-coding RNA and *Fzd10* and *Gigyf2* encode signalling molecules. No information is available in databanks on the predicted protein encoded by *Wdr60*.

To validate the expression profiles obtained by microarray analysis, real-time PCR was used to compare the mRNA expression levels in compressed and control chondrocytes. We examined eight genes and confirmed the same gene expression modulation pattern as the microarray analysis (Figure 4, panel B). These observations indicated that our experimental procedure reliably identified putative mechanosensitive genes.

In addition, the microarray analysis revealed many other candidate mechanosensitive genes when the fold change threshold was lowered from 2 to 1.4 (p<0.01, Data S2). This extended dataset included 325 genes, with 48 up-regulated (i.e. 15%) and 277 down-regulated genes (i.e. 85%). The early response of chondrocytes to compression is thus generally characterised by down-regulation of gene expression (Figure 5 Panel A).

The presence of numerous transcription factors on the short list of highly responsive genes suggests that our cell model system was suitable for exploring the early events of mechanotransduction. We sought to further confirm this hypothesis by using the extended dataset of mechanosensitive genes. Hence, we analysed this extended list using PANTHER classification system to cluster candidate genes into relevant categories regarding signal transduction. From the extended dataset, 212 coding transcripts were eligible for functional annotation, of which 41 were up-regulated and 171 were down-regulated proteins (Data S3). Transcription factors were the most over-represented class of proteins (36 proteins, p<0.001) and when pooled with DNA-, RNA- and nucleic acid-binding proteins (63 proteins), they represented 30% of the modulated proteins detected here (Figure 5 Panel B). In addition, 20 proteins belonged to the protein class grouping kinases, phosphatases and kinase regulators, and 25 proteins belonged to the protein class grouping receptors and receptor-binding proteins (Figure 5 Panel B and Data S4). Since these data strongly suggest that chondrocytes are involved in signal transduction mechanisms, the dataset of the 212 functionally annotated proteins was further analysed using the PANTHER and Pathway Express systems to identify over-represented signalling pathways. Several signalling pathways, such as Wnt, TGF-β, or MAPK pathways, were prominent, although statistical support was modest (data not shown). Altogether, our results demonstrate the relevance of the extended list of modulated genes for identifying new actors or targets involved in chondrocyte mechanotransduction.

## Discussion

### Validation of the chondrocyte-agarose construct as a model for identifying the mechanosensitive response typical of chondrocytes

The aim of this study was to explore the molecular-level response of chondrocytes to dynamic compression using a model system we previously developed [8], [9]. Because sensing and response to external mechanical stimuli by cells is controlled by cell-matrix interactions, we carefully examined — before performing the compression experiments — the extracellular matrix proteins and cellular receptors synthesised by chondrocytes in agarose. Western blot analysis extended our previous immunohistochemistry studies [8], [9] and confirmed that chondrocytes produced a cartilage-characteristic matrix during the pre-culture period. Regarding type II and type IX collagen production, the presence of cross-links in the newly formed matrix indicated that these chondrocytes were able to synthesise enzymes necessary for proper maturation and stabilisation of collagen molecules and their packing into collagen fibrils. In addition, in our model system, chondrocytes expressed the collagen-binding integrin α10 [23]. Therefore, the chondrocyte-agarose model system used in this study made it possible to examine the molecular events underlying mechanotransduction, which probably occur during typical chondrocyte-cartilage matrix interactions.

Since chondrocyte response to mechanical stimulation is affected if chondrocytes dedifferentiate prior to compression [24], [25], we also carefully examined the chondrocyte phenotype in our model system. Western blot analysis of type I, II and IX collagens, α10 and α11 integrins and Sox9 extends our previous studies [8], [9] and confirmed that chondrocytes maintain a well-differentiated phenotype in our model system. Agarose hydrogel cultures have already been used to enhance chondrocytes in other models [26], [27]; the challenge here was to use freshly isolated mouse cells and to obtain a complete differentiated phenotype after one week of culture.

### Suitability of our model system for studying the early events of mechanotransduction

In the chondrocyte-agarose constructs, the levels of phospho-FAK were not significantly different in compressed compared to uncompressed cells, contradicting numerous published results showing a rapid activation of FAK following various mechanical stimuli. One possible explanation is that, in contrast to our chondrocyte-agarose constructs, cells where not embedded in a 3D environment. It is well known that cells in 3D systems form matrix adhesions that are not the same as their 2D counterparts [28].

We detected transient activation of ERK1/2 and p38 in response to mechanical stress, as expected from previous studies [13]–[15]. We also found that *Fos* and *Jun* family members and *Egr-1* gene expressions were activated after 30 min of compression, shortly after the primary activation of the MAPK pathway. These results are very consistent since *Fos*, *Jun* and *Egr-1* are downstream targets of the MAPK pathway activated by compression in chondrocytes [20], [29]. In addition to *Fos*, *Fosb*, *Jun* and *Junb*, the *Atf3* gene was also stimulated 1.47-fold by compression (Data S2). This modulation is in good agreement with the modulation observed for AP-1 genes since Atf3, a transcription factor known to be induced in stress responses, forms heteromers with Jun members for its transcriptional activities [30].

Overall, the microarray analysis revealed that very few gene expression levels were modulated more than 2-fold, suggesting that dynamic compression triggered modest regulatory events. All the up-regulated genes in this list are already known as “immediate early genes”. Examination of the very early events of dynamic compression reduces the risk of interpreting the result of feedback signalling. The presence of numerous transcription factors among the 20 most responsive genes was consistent with a high frequency of genes with a >1.4-fold change in expression that code for proteins linked to signal transduction and gene expression regulation. These results further demonstrate that our model system is useful for studying mechanotransduction early events.

### Characteristic TGF-β signalling is activated by dynamic compression

Only a few studies have reported activation of TGF-β/Smad signalling as an early event in cellular mechanotransduction. Osteoblasts and the Saos-2 osteoblastic cell line respond to mechanical stimulation by increasing the activation of bone morphogenetic protein (BMP) receptor substrates, Smad1/5 [31]–[33]. Likewise, Smad2/3 phosphorylation increases when umbilical cord progenitor cells are stretched [34]. Regarding chondrocytes, only one immunohistochemistry study has shown Smad2/3 activation in specific regions of bovine articular cartilage subjected to 5 min of shear stress [17]. In our study, a Western blot analysis showed that Smad2, but not Smad1/5/8, was activated by dynamic compression, thus confirming that activation of TGF-β/Smad signalling represents an early response of chondrocytes to mechanical loading.

Chondrocytes cultured in agarose gel secrete TGF-β [35] and this protein is secreted by various cells — including chondrocytes — as part of a latent complex that associates with matrix proteins such as fibrillin, proteoglycans, and fibronectin [36]–[38]. One component of the latent complex, the latency-associated protein, interacts directly with integrins, especially αvβ5. Myofibroblasts cultured on stiff matrices can exert tension on the latent complex through integrins, causing conformational changes and the release of sequestered TGF-β in an active form [39]. Although we did not measure the release of active TGF-β, it is possible that dynamic compression on chondrocyte-agarose constructs causes the mechanically driven release of soluble TGF-β which then binds to its receptor and subsequently triggers signalling as exemplified by Smad2 phosphorylation.

Regardless of the exact mechanism of TGF-β activation in our cell model system, the microarray analysis confirmed the involvement of TGF-β signalling in the chondrocyte response to dynamic compression. For instance, *Htra1*, a gene coding for a serine protease that inhibits TGF-β signalling [40] and *Arkadia/Rnf111*, a gene coding for an ubiquitin ligase involved in Smad2/3 regulation [41], were down-regulated under dynamic compression (1.47-fold and 1.66-fold, respectively, Data S2). Moreover, *Cyr61* was up-regulated by 1.64-fold. *Cyr61* is an important regulator of chondrogenesis and a member of the CCN family that includes connective tissue growth factor (Ctgf) [42]. *Cyr61*, like *Ctgf*, is up-regulated in fibroblasts cultured under mechanical stress within a 3D collagen gel [43]. Because *Cyr61* expression is activated as an early response to TGF-β [44], it is possible that the observed *Cyr61* up-regulation results, at least in part, from the activation of TGF-β signalling triggered by dynamic compression. Clearly, the interplay between growth factors, growth factor signalling and mechanotransduction is highly complex.

### Dynamic compression induces a general down-regulation of gene expression in chondrocytes

The microarray analysis revealed that around 85% of the 325 mechanosensitive identified genes were down-regulated. One possible explanation for the observed trend towards a reduction in RNA levels is an increase in mRNA decay. Interestingly, two genes coding for major proteins involved in RNA degradation, *Btg2* and *Zfp36*, were one of the relatively few genes up-regulated following dynamic compression (2.28-fold and 1.76-fold, respectively, Data S2). Btg2, a member of the Btg/Tob family of proteins, is a general activator of mRNA decay [45], and Zfp36 binds to unstable mRNA and promotes their degradation [46]. Zfp36 has been proposed as an inducible attenuator of growth factor signalling, by promoting degradation of rapidly induced genes and thus restricting the cell's responsiveness to stimulation [47]. Btg/Tob factors are thought to facilitate the rapid switch to a new gene expression program by speeding up the degradation of previously made mRNAs [45]. For example, Btg2 activates BMP signalling [48]. Down-regulation of gene expression may therefore represent a general mechanism in the early response of chondrocytes to mechanical stress.

### Dynamic compression affects various aspects of chondrocyte physiology

Independently of the general down-regulation observed in gene expression, careful examination of the extended list of modulated genes indicates that some of them have already been identified as mechanosensitive genes involved in different aspects of cellular physiology like in cartilage, e.g. *Biglycan (Bgn)*, an extracellular matrix protein [49], *Mmp9*, a matrix metalloprotease [50], *Cyr61*, a regulator of chondrogenesis from the CCN family [43], *Cited2*, a transcription co-regulator playing a key role in shear-induced regulation of MMPs in chondrocytes [51], or in other tissues, e.g. *Thrombomodulin (Thbd)*, a gene coding for an anticoagulant factor [52], *Lmo4*, a fluid flow-responsive transcription factor [53], *Ptgs1/Cox1*, a cyclooxygenase involved in the production of prostaglandin E2 [54], or *Ahnak*, a protein involved in Ca^2+^ signalling pathways and regulated exocytosis [55], [56]. The regulation of these genes reflects diverse cellular responses to mechanical stimulation.

Interestingly, a subset of modulated genes, including *Pcm1* [57], *Nek1* [58], *Smo* [59], *Cdk5rap2* [60], *Spop* [61], *Dync2h1* [62], *Syne1/Nesprin1* [63], *Topors* [64] and Wnt signalling molecules [65] such as *Fzd10, Sfrp1, Rspo3/Cristin1*, are linked to ciliary function (Data S2). The primary cilium has long been hypothesised to function as an antenna for chondrocytes to sense the biomechanical environment, as in renal cells [66], [67]. Using the same chondrocyte-agarose constructs as those used here, Wann *et al.* have just provided the first direct experimental evidence that the primary cilium mediates mechanotransduction through control of calcium signalling in compressed chondrocytes [68]. Previously, using bovine chondrocyte-agarose constructs and confocal microscopy, McGlashan *et al.* showed that the application of cyclic compression affects cilia length in a time-dependent manner [69]. In addition, mechanical forces have been reported to play a role in primary cilia assembly/disassembly *in vitro* in other cell types [70], [71]. These observations are correlated with *in vivo* studies, where the presence or absence of cilia is linked to the intensity of shear stress in blood vessels [72]. Therefore, the mechanosensitivity observed here for the subset of cilium-related genes may represent an early signal triggered by chondrocytes to adapt the length and/or function of the primary cilium in response to mechanical loading.

Nevertheless, part of the RNA transcriptome corresponds to RNAs that do not code for proteins, referred to as non-coding RNAs (ncRNAs) [73]. Microarray screening identified two down-regulated long ncRNAs in compressed chondrocytes: *Xist* and *Malat1* (1.57-fold and 1.42-fold, respectively, Data S2), which are two of the three large non-coding transcripts present in mammalian nuclei [74]. Furthermore, *Dicer1*, a gene coding for an endoribonuclease that processes pre-miRNAs into siRNAs [75], was down-regulated by 1.81-fold upon compression. In particular, recent studies have shown that miRNA can control expression of alternative splicing regulators [76] and *Malat1* can control the activity of some miRNAs [77]. This is particularly interesting because alternative splicing events have been recorded in bone following mechanical loading [78]. These findings suggest that modulation of ncRNA expression is part of the molecular response to mechanical stress. Moreover, it is possible that these ncRNAs participate in the regulation of pre-mRNA splicing in response to compression.

### Concluding remarks

The aim of this study was to perform an integrated analysis of mechanotransduction in chondrocytes at the gene and protein level. The originality of our analysis was to investigate early molecular events triggered by dynamic compression. Our study reveals that, in addition to the well-known involvement of the MAPK-signalling pathway in the chondrocyte mechanotransduction response, TGF-β signalling may also play a prominent role. In addition, our microarray analysis results provide new molecular insight into how chondrocytes sense dynamic compression. The candidate mechanosensitive genes identified here can serve as starting points for future investigations of mechanotransduction in chondrocytes.

The availability of genetically modified mice offers an opportunity to study the impact of gene modification in chondrocyte mechanotransduction using the cell model system presented here. Ultimately, identifying candidate mechanosensitive genes can provide important information not only for the molecular understanding of mechanotransduction in chondrocytes, but also for cartilage engineering. For example, agarose (or agarose-alginate) hydrogels constitute clinically potential scaffolds for autologous chondrocyte implantation [79] and mechanical conditioning can be used to stimulate *in vitro* chondrocyte biosynthesis in 3D scaffolds before implantation. Therefore, mechanosensitive targets can help optimise mechanical conditioning for cartilage reconstruction.

## Materials and Methods

### Ethics statement

Mouse care and treatment were conducted in accordance with institutional guidelines in compliance with national and international laws and policies. This study was specifically approved by our local ethics committee (Authorization n°69387416 given by the French Prefecture du Department du Rhone).

### Antibodies

For type I and type II collagens, polyclonal rabbit antibodies against mature collagens were used (Novotec; references 20151 and 20251, respectively; used at 1∶2000 and 1∶5000, respectively). Monoclonal antibody (mAb) against collagen IX (23-5D1; 1∶6000) was a gift from Bjorn Olsen (Boston, MA). Polyclonal antibodies against α10 (1∶2000) and α11 (1∶4000) integrins were from Cartela AB (a gift from Evy Lundgren-Akerlund, Lund, Sweden). Antibodies against Phospho-Smad1/5/8 (#9511), Phospho-Smad2 (#3101), Phospho-ERK1/2 (#9101), Phospho-p38 (#9251), Smad2/3 (#3102), ERK1/2 (#9102), p38 (#9212) and anti-rabbit IgG horseradish peroxidase (HRP)-linked antibodies were purchased from Cell Signaling Technology (all 1∶1000). Rabbit mAb to Smad1 (1649-1) and Smad5 (1682-1) were from Epitomics and used both 1∶1000 in mixture. Anti-Sox9 polyclonal antibody (AB5535, 1∶2000) and anti-FAK monoclonal antibody (clone 4.47, 1∶5000) were purchased from Millipore. Polyclonal rabbit antibodies against phosphoY397-FAK (1∶1000) were obtained from Biosource-Invitrogen. Anti-actin monoclonal antibodies (A5060, 1∶800) were purchased from Sigma-Aldrich. Anti-mouse (170-6520) or rabbit (170-6518) IgG-alkaline phosphatase conjugates and anti-mouse IgG-HRP conjugates (170-6516) were purchased from Bio-Rad, all used 1∶5000.

### Chondrocyte isolation and 3D culture

Embryonic mouse chondrocytes were isolated from the costal cartilage of day 17.5 post-coitum mice. Like articular cartilage, rib cartilage is a hyaline-type cartilage. Immediately after enzymatic isolation, cells were embedded in 2% agarose gels at a density of 2×10^6^ cells/mL as described [9]. Chondrocyte-agarose gels were punched to form cylindrical constructs of 13 mm in diameter and 3 mm in thickness. They were then cultured in the wells of Biopress™ compression plates (Flexcell international) for 6 days in 5% CO_2_ at 37°C. The Dulbecco's modified Eagle's medium/Ham's F-12 culture medium was changed daily as previously detailed [9]. Serum was progressively substituted with insulin-transferrin-selenium and cultures were gradually supplemented with ascorbic acid (up to 20 µg/mL). Used as positive controls of dedifferentiation, other mouse chondrocytes were cultured in monolayer for one week, passaged once and cultured for another week.

### DNA content

DNA quantification was performed using the Hoechst 33258 (Fluka) DNA stain. The calibration curve was obtained using a DNA standard solution (Invitrogen).

### Application of dynamic compression

Chondrocyte-agarose constructs were subjected to compression using a previously characterised model system [9], [8]. The FX-4000C Flexercell Compression Plus System (Flexcell International) was used to apply dynamic compressive strain to agarose gels. Compressed constructs were subjected to cyclical compression ranging from 20 kPa to 40 kPa in a square waveform at a frequency of 0.5 Hz (Figure 2) for 5, 15 or 30 min. Control constructs were uncompressed.

### Protein extraction and analysis by Western blotting

Protein extraction from the agarose gels was performed with special care to avoid any modification in the phosphorylation state of proteins [9]. For Western blotting, proteins were separated on 10% or 4–12% polyacrylamide gradient mini-gels and transferred to PVDF membranes (Millipore). The membranes were probed with the appropriate primary antibodies, washed and incubated with HRP- or alkaline phosphatase-conjugated anti-mouse or anti-rabbit IgG. After multiple washes, bound antibodies were detected on x-ray films using a Bio-Rad Immun-star or WesternC chemiluminescent substrate. The membranes probed with antibodies to collagens or integrins were sequentially re-probed after stripping (Re-Blot Plus Strong, Chemicon). A final re-probing with anti-actin antibodies served as a loading control. The membranes probed with antibodies to phospho-proteins were stripped and re-probed with antibodies that recognise all forms of the protein in question. Phosphorylation levels were quantified by densitometry using ImageQuant software (Molecular Dynamics). For each protein, the ratio of phospho-protein band intensity to the total protein band intensity was calculated and mechanically-induced phosphorylation was normalised to uncompressed controls.

### DNA microarray analysis

Total RNA was extracted from chondrocyte-agarose constructs as previously described [9]. To ensure a sufficient quantity of RNA, extractions from six similar constructs were pooled. To ensure quality of RNA in each sample, integrity and purity were assessed using a capillary electrophoresis system (Agilent Bioanalyser, Agilent Technologies). DNA microarray analysis was performed on four independent experiments to compare gene expression levels between compressed (30 min compression) and uncompressed (control) constructs.

Hybridisation was carried out following the Two-Colour Microarray-Based Expression Analysis protocol (Agilent Technologies) and 500 ng or 1 µg of purified total RNA were used for linear amplification. The resulting labelled cRNA from a compressed sample was co-hybridised with the labelled cRNA of the corresponding control sample to the Agilent Mouse Genome CGH Microarray 44 K probe set (Agilent Technologies). Each co-hybridization was performed several times starting from different total RNA preparations and using a dye swap. Each microarray contained 44,000 sequences spanning the whole mouse genome and control probes. The microarrays were scanned using an Innoscan 700 Microarray Scanner (Innopsys) at 532 nm (for detection of the Cy3 dye) and 635 nm (Cy5 dye). The resulting image was analysed using Mapix v3.1 software. The signal intensity of each spot was acquired and non-exploitable spots were filtered out.

The statistical analysis and normalisation steps were done using the Limma (Linear Models for Microarray Data) package [80] in the statistical language R [81]. The “global Loess” function was applied to the data to correct for bias. Normalised data were then averaged between direct and swapped comparisons to calculate values of differential expression and expression level. A classification of statistically significant modulations was obtained using a moderated Student's *t*-test with a Bayesian false-discovery rate approach [82]. Analysis of genes associated with cell function was carried out using the PANTHER (Protein ANalysis THrough Evolutionary Relationships) classification system (http://www.pantherdb.org) and Pathway-Express (http://vortex.cs.wayne.edu/Projects.html) profiling system to identify protein categories or biological pathways which may be associated with modulated gene expression (with M>0.5 and p<0.01).

### Confirmation of modulation in gene expression by real-time PCR

Real-time PCR analysis was performed on three independent experiments as previously described [9]. Levels of gene expression were determined by using the comparative Ct method with *RPL13a* gene as the endogenous control. Primer pairs used in this study are described in Table 2. Dissociation curves were conducted at the end of each run to verify the absence of DNA contamination. Student's *t*-test (paired, two-tailed) was used for statistical analysis.

## Supporting Information

Data S1 ERK1/2 and p38 transient compression-induced activation in chondrocyte-agarose constructs. Legend: Chondrocytes cultured in agarose for 6 days underwent dynamic compression (+) or were not compressed (−) for the indicated times and phosphorylation levels of ERK1/2 and p38 were analysed on Western blots. (A) Representative blots. (B) Densitometric analysis was performed on four (5 and 15 min) or three (30 min) independent experiments. The phospho-MAPK to total MAPK ratio was calculated and mechanically induced phosphorylation was normalised to uncompressed controls. Bars represent the compression-induced phosphorylation modulation (mean fold change +/− SD), either up-regulation (red) or down-regulation (green) (* p<0.05).
(TIF)

Data S2 Results from DNA microarray analysis: gene expression levels in compressed samples were compared to uncompressed control samples (fold change >1.4 and p-value >0.01).
(XLS)

Data S3 Results from DNA microarray analysis: modulated coding transcripts eligible for functional annotation (PANTHER analysis).
(XLS)

Data S4 Results from DNA microarray analysis: modulated coding transcripts sorted by protein class (PANTHER analysis).
(XLS)
