# Supplementary figures and images for: Dynamic Compression of Chondrocyte-Agarose Constructs Reveals New Candidate Mechanosensitive Genes
Source: PLoS One. 2012 May 17;7(5):e36964. doi: 10.1371/journal.pone.0036964 (PMC3355169; doi:10.1371/journal.pone.0036964)

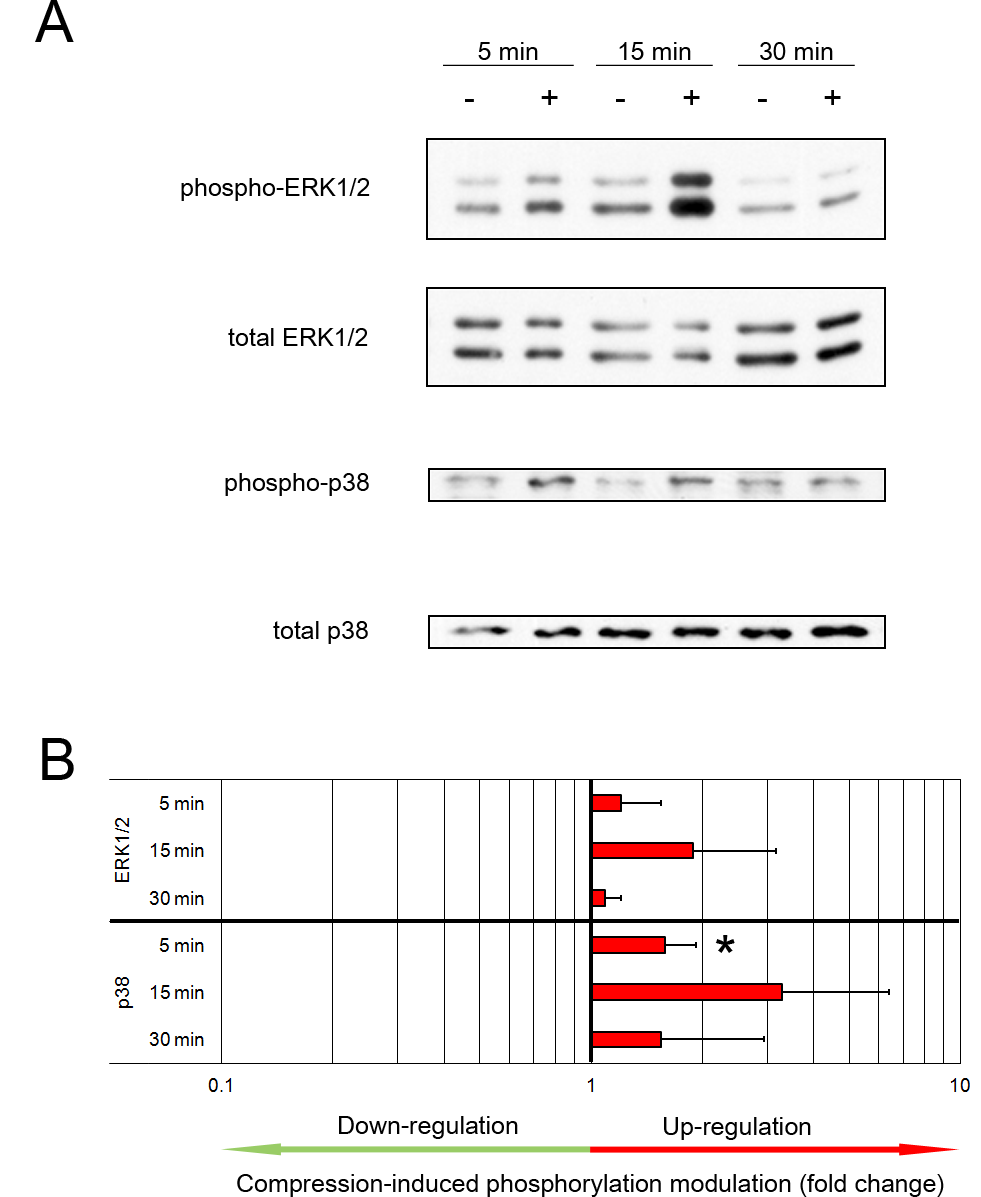

Supplement: Data S1 — ERK1/2 and p38 transient compression-induced activation in chondrocyte-agarose constructs. Legend: Chondrocytes cultured in agarose for 6 days underwent dynamic compression (+) or were not compressed (−) for the indicated times and phosphorylation levels of ERK1/2 and p38 were analysed on Western blots. (A) Representative blots. (B) Densitometric analysis was performed on four (5 and 15 min) or three (30 min) independent experiments. The phospho-MAPK to total MAPK ratio was calculated and mechanically induced phosphorylation was normalised to uncompressed controls. Bars represent the compression-induced phosphorylation modulation (mean fold change +/− SD), either up-regulation (red) or down-regulation (green) (* p<0.05). (TIF) [file pone.0036964.s001.tif]
